# Virtual and Augmented Reality Applications in Medicine: Analysis of the Scientific Literature

**DOI:** 10.2196/25499

**Published:** 2021-02-10

**Authors:** Andy Wai Kan Yeung, Anela Tosevska, Elisabeth Klager, Fabian Eibensteiner, Daniel Laxar, Jivko Stoyanov, Marija Glisic, Sebastian Zeiner, Stefan Tino Kulnik, Rik Crutzen, Oliver Kimberger, Maria Kletecka-Pulker, Atanas G Atanasov, Harald Willschke

**Affiliations:** 1 Oral and Maxillofacial Radiology, Applied Oral Sciences and Community Dental Care Faculty of Dentistry The University of Hong Kong Hong Kong China; 2 Ludwig Boltzmann Institute for Digital Health and Patient Safety Medical University of Vienna Vienna Austria; 3 Department of Molecular, Cell and Developmental Biology University of California Los Angeles Los Angeles, CA United States; 4 Division of Pediatric Nephrology and Gastroenterology, Department of Pediatrics and Adolescent Medicine Comprehensive Center for Pediatrics Medical University of Vienna Vienna Austria; 5 Swiss Paraplegic Research Nottwil Switzerland; 6 Institute of Social and Preventive Medicine University of Bern Bern Switzerland; 7 Department of Anaesthesia, Intensive Care Medicine and Pain Medicine Medical University Vienna Vienna Austria; 8 Ludwig Boltzmann Institute for Digital Health and Prevention Salzburg Austria; 9 Department of Health Promotion, Care and Public Health Research Institute (CAPHRI) Maastricht University Maastricht Netherlands; 10 Institute for Ethics and Law in Medicine University of Vienna Vienna Austria; 11 Institute of Genetics and Animal Biotechnology of the Polish Academy of Sciences Jastrzebiec Poland; 12 Institute of Neurobiology Bulgarian Academy of Sciences Sofia Bulgaria; 13 Department of Pharmacognosy University of Vienna Vienna Austria

**Keywords:** virtual reality, augmented reality, mixed reality, bibliometric, surgical procedures, rehabilitation, neurodegenerative disorder, pain, stroke, medicine

## Abstract

**Background:**

Virtual reality (VR) and augmented reality (AR) have recently become popular research themes. However, there are no published bibliometric reports that have analyzed the corresponding scientific literature in relation to the application of these technologies in medicine.

**Objective:**

We used a bibliometric approach to identify and analyze the scientific literature on VR and AR research in medicine, revealing the popular research topics, key authors, scientific institutions, countries, and journals. We further aimed to capture and describe the themes and medical conditions most commonly investigated by VR and AR research.

**Methods:**

The Web of Science electronic database was searched to identify relevant papers on VR research in medicine. Basic publication and citation data were acquired using the “Analyze” and “Create Citation Report” functions of the database. Complete bibliographic data were exported to VOSviewer and Bibliometrix, dedicated bibliometric software packages, for further analyses. Visualization maps were generated to illustrate the recurring keywords and words mentioned in the titles and abstracts.

**Results:**

The analysis was based on data from 8399 papers. Major research themes were diagnostic and surgical procedures, as well as rehabilitation. Commonly studied medical conditions were pain, stroke, anxiety, depression, fear, cancer, and neurodegenerative disorders. Overall, contributions to the literature were globally distributed with heaviest contributions from the United States and United Kingdom. Studies from more clinically related research areas such as surgery, psychology, neurosciences, and rehabilitation had higher average numbers of citations than studies from computer sciences and engineering.

**Conclusions:**

The conducted bibliometric analysis unequivocally reveals the versatile emerging applications of VR and AR in medicine. With the further maturation of the technology and improved accessibility in countries where VR and AR research is strong, we expect it to have a marked impact on clinical practice and in the life of patients.

## Introduction

Virtual reality (VR) is a technology that immerses the user in a synthetic 3-dimensional (3D) environment via wearable screens in the form of VR headsets, while closely related augmented reality (AR) uses elements of VR and superimposes them on to the real-world environment in the form of a live video displayed on the screen of an electronic device [1]. VR is a concept that has been developing over the last 50 years, whereas AR is a relatively new concept. Both are aimed at providing an experience for the users that engages their visual and auditory senses by creating an illusion of the surroundings [2,3]. In recent years, and with the advancement of technology, both VR and AR systems have become more portable, more realistic, and better to navigate in real time, adding a sensory and sometimes olfactory element to the range of sensations [3]. Furthermore, head-mounted devices are becoming more accessible. Personalized VR and AR devices have already been on the market for several years and are constantly improving and developing. The user can interact with the virtual environment through hand-held devices such as joysticks or keyboards and more recently, using integrated body tracking technologies [2,3]. VR and AR can be very versatile, using different systems and setups as well as different content that can range from very immersive, dynamic, and interactive to nonimmersive and static. Immersion, presence, and interaction are 3 essential characteristics of VR and AR [2,4]. Immersion is dependent on the technology used; it can be a head-mounted device, concave or 3D projection, or video where the user is the protagonist. Presence and interaction are related to the individual perception of being connected to the environment and the ability to act within the environment and receive feedback and reaction.

VR and AR are digital technologies that allow automation and can be used in fields where repetitive tasks need to be performed and often perfected [2]. A common example for the use of VR and AR in the medical field is medical education and training, especially in surgery [5-7]. For example, using VR or AR in surgical procedure training allows the trainee to perform steps on a virtual patient or having patient information superimposed with reality [1,5,7]. There is some evidence that VR could be a useful tool in improving surgical skills and reducing surgical procedure errors [8]. Neurosurgery, representing a traditionally complex surgical area, has been positively affected by the development of virtual techniques [9]. In acute pain management, VR has been used as a distraction technique [10,11], and there are studies proposing a role of VR in chronic pain management by inducing neurophysiological changes beyond simple distraction [10,12,13]. Further interesting prospects are using VR technology for the treatment of “phantom limb pain” following amputation [14] or after spinal cord injury [15]. VR has been used in rehabilitation for improvement of upper limb function following stroke [4], with modest or no improvement over conventional physiotherapy. Some benefits could be exhibited, on the other hand, in improving the cognitive abilities of patients with stroke, in particular speech, attention, and memory [16]. VR can also benefit patients with mental health conditions such as anxiety, depression, substance abuse, or eating disorders [17] and has been used as a therapy in a number of phobias and posttraumatic stress disorder [18].

The benefits of using VR or AR over conventional therapy could be plentiful: It might allow for multiple repetitions of simple tasks in clinical practice in an immersive environment without the need for constant supervision by medical staff, which could considerably reduce the costs for training facilities and trained medical staff. Furthermore, and especially for immobile patients, head-mounted devices could be safely used in patients’ homes, which could decrease the need for hospital visits. VR and AR experiences can be designed to be attractive and user-friendly, decreasing the attrition rate of patients and providing a more pleasurable environment. From a research perspective, the use of VR can facilitate data collection for monitoring of progress [19]. Using VR in surgical training could vastly reduce the possibility for surgical errors, leading to great improvement in patient safety [8].

While the number of studies on the use of virtual technologies in health care is growing, these studies tend to be small and heterogeneous and often lack proper controls [4]. Results from such studies are often inconclusive, and the benefits of virtual over conventional approaches in health care are difficult to determine. The mechanisms by which VR treatment provides pain relief, for example, are still debated [12]. Moreover, a long-term benefit of VR treatment, especially in chronic pain management, has yet to be established. In a clinical setting, establishing VR systems is still technically challenging and cost-prohibitive, and such technology often has lower levels of acceptance in the elderly population [2]. A comprehensive guideline for standardization of the use of these technologies in medicine is still lacking and warrants further consideration.

Bibliometrics is an analytical approach that generates an integrative view and quantitative parameter profiling of entire research fields or specific scientific application areas [20-22]. Previous work has focused on specific areas of VR application such as dementia and rehabilitation medicine [23,24]. For instance, highly cited papers describing VR application for autism spectrum disorder (ASD) focused their research mainly on the improvement of social skills [23], whereas research in the context of dementia mainly focused on the application of VR as an assessment tool for spatial navigation, memory profile, memory deficit, and memory formation in patients with mild cognitive impairment [24]. As VR is becoming a more prevalent topic in health care and medicine, we aimed to conduct a bibliometric analysis of the current literature, to discover trends and topics explored in VR applications in medicine and quantitatively evaluate the available literature. To the best of our knowledge, this is the first total-scale bibliometric analysis examining overall VR and AR applications in medicine in the scientific literature.

## Methods

### Data Source and Search Strategy

The Web of Science (WoS) Core Collection database was searched on September 16, 2020 and queried with the following search string: TOPIC: (“virtual reality*” OR “augmented reality*” OR “mixed reality*” OR “computer-mediated reality*”) AND TOPIC: (medic* OR illness* OR disease* OR health* OR pharma*). The search identified publications mentioning these words and their derivatives in the title, abstract, or keywords. No additional restrictions, such as publication type or language, were used. The “Analyze” and “Create Citation Report” functions of WoS were utilized for basic publication and citation counting. The full records of the resultant publications were exported to VOSviewer (version 1.6.15) and Bibliometrix (Version 3.0 operated under the web interface called Biblioshiny) for further bibliometric analyses.

VOSviewer was used to produce a term map showing phrases from titles and abstracts of the publications. For clarity, phrases occurring in at least 0.5% (42/8399) of the publications were included. Multiple appearances in a single publication counted as one. In the obtained map, the circle size represents the frequency of occurrence, whereas the color represents the citations per publication. The distance between 2 circles represents how 2 phrases co-occurred with each other in the publications. Meanwhile, a density map was produced to show author´s keywords in the publications. For clarity, keywords occurring in at least 0.1% (9/8399) of the publications were included. A keyword or cluster of keywords with higher frequency counts formed a red region, and those with lower frequency counts formed a yellow region.

Utilizing the software Biblioshiny, the Trend Topics function was used to reveal trends in abstract words. Words were included if they occurred in at least 5 publications. Each year was limited to 5 words.

### Statistical Analysis

To analyze citation per publication (CPP) differences between original articles and reviews as well as between core and noncore journals, a 2-sample *t* test was conducted. In order to assess differences in CPP between different research areas (as designated by WoS), a one-way analysis of variance was utilized. Statistical tests were performed with SPSS (version 26.0; IBM Corp, Armonk, NY). Results were deemed significant if *P*<.05.

## Results

### Overall Literature Landscape

**Figure 1:**
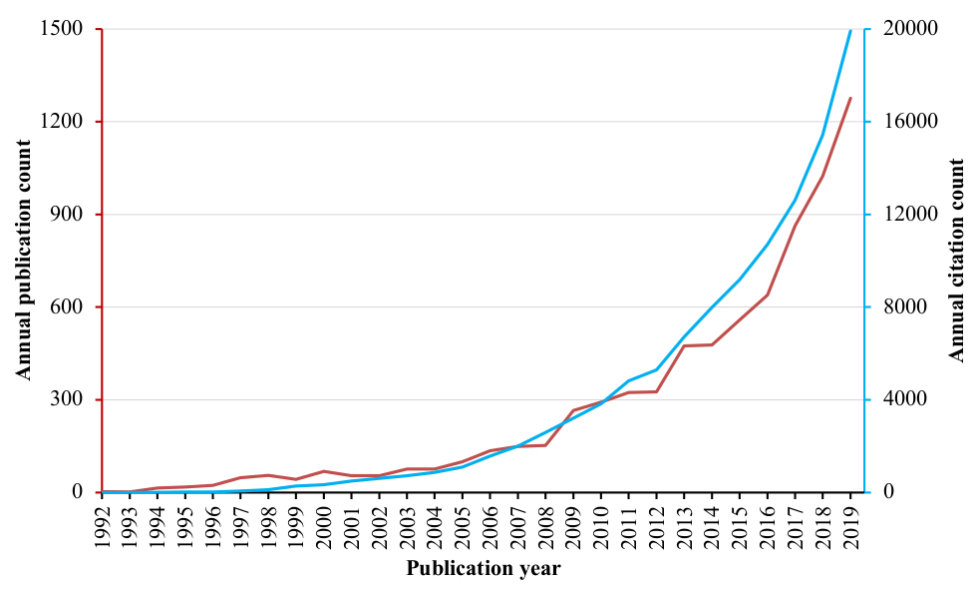
Annual publication and citation count of virtual reality research in medicine.

The literature search identified 8399 publications published from 1992 until 2020. The annual publication count reached 100 since 2005 and exceeded 1000 since 2018 (Figure 1). The year 2020 had 802 published papers until the date of the literature search (9/16/2020). There were 5297 original articles (5297/8399, 63.07%; CPP=17.4) and 902 reviews (902/8399, 10.74%; CPP=31.1). Therefore, the article-to-review ratio was 5.9:1, with reviews having a significantly higher CPP than original articles (*P*<.001). Other major document types were proceedings papers (1908/8399, 22.72%; CPP=2.0; WoS tagged 2213, of which 305 were simultaneously tagged as original articles), editorial materials (135/8399, 1.61%; CPP=13.7), and meeting abstracts (130/8399, 1.55%; CPP=0.2). The analyzed papers were mainly published in English (8131/8399, 96.81%).

### Most Productive Entities

The 10 most productive authors by number of publications are listed in Table 1. The most productive author was Professor Giuseppe Riva from General Psychology and Communication Psychology, Catholic University of Milan, Italy. He also had the highest H-index among the 10 most productive authors. Meanwhile, the author with the highest CPP was Professor Albert “Skip” Rizzo from Medical Virtual Reality, Institute for Creative Technologies, University of Southern California. From the M-index, it was noted that among these authors, the most impactful newcomer to the field was Dr Silvia Serino from MySpace Lab, Department of Clinical Neurosciences, University Hospital Lausanne, Switzerland.

The 10 most productive organizations are listed in Table 2; 5 of these were in the United States, 2 each in the United Kingdom and Italy, and 1 in Canada. The University of London was the most productive organization, and the University of Toronto had the highest CPP, whereas the earliest research in this field was published at Harvard University.

**Table 1:** The 10 most productive authors of virtual reality research in medicine (8399 articles).

Author	Number of publications, n (%)	Citations per publication (CPP)	H-index^a^	M-index (first year)^a,b^
Giuseppe Riva	114 (1.36)	18.5	27	1.29 (2000)
Brenda K. Wiederhold	63 (0.75)	15.0	15	0.71 (2000)
Nassir Navab	60 (0.71)	12.6	16	0.76 (2000)
Albert “Skip” Rizzo	50 (0.60)	32.1	20	0.95 (2000)
Cristina Botella	37 (0.44)	17.0	16	0.80 (2001)
Mariano Alcaniz	36 (0.44)	22.7	15	0.75 (2001)
Pietro Cipresso	34 (0.40)	12.2	11	1.10 (2011)
Andrea Gaggioli	34 (0.40)	17.2	10	0.50 (2001)
Silvia Serino	33 (0.39)	14.6	13	1.63 (2013)
Lars Konge	32 (0.38)	13.8	12	1.20 (2011)

^a^Calculated from the dataset.

^b^Calculated by dividing the H-index by the number of years since the first published paper (within the dataset) of the author.

**Table 2:** The 10 most productive organizations of virtual reality research in medicine (8399 articles).

Organization	Publications, n (%)	Citations per publication (CPP)	H-index^a^	M-index (first year)^a,b^
University of London	175 (2.08)	27.5	37	1.61 (1998)
University of California System	164 (1.95)	22.4	34	1.42 (1997)
Harvard University	140 (1.67)	24.6	30	1.03 (1992)
University of Toronto	132 (1.57)	42.5	34	1.55 (1999)
Imperial College London	124 (1.48)	27.8	35	1.30 (1994)
Istituto Auxologico Italiano	116 (1.38)	18.1	27	1.29 (2000)
University of Southern California	113 (1.35)	29.1	29	1.07 (1994)
Catholic University of the Sacred Heart	112 (1.33)	17.5	25	1.19 (2000)
State University System of Florida	107 (1.27)	25.2	22	0.88 (1996)
Pennsylvania Commonwealth System of Higher Education	103 (1.23)	19.6	25	1.09 (1998)

^a^Calculated from the dataset.

^b^Calculated by dividing the H-index by the number of years since the first published paper (within the dataset) of the author.

The 10 most productive countries are listed in Table 3. The United States had contributions to nearly 30% of the VR publications in medicine and had the highest CPP. The international collaboration rates of these countries were mostly around 20%-30%, with the United States having a lower rate at 12.3%.

The 10 most productive journals are listed in Table 4. Computer science, surgery, and psychology were the 3 major research areas of these productive journals. Among the list, computer science journals seemed to have much lower CPP compared to others. According to Bradford’s law, core journals are defined as the most productive journals that collectively account for publishing one-third of all concerned articles [25]. By this definition, there were 85 core and 3565 noncore journals in this dataset. The CPP of core (14.8) and noncore (15.0) journals did not significantly differ (*P*=.845).

**Table 3:** The 10 most productive countries of virtual reality research in medicine (8399 articles).

Country	Publications, n (%)	Citations per publication (CPP)	SCP^a^	MCP^b^ (% of MCP:MCP+SCP)^c^
United States	2457 (29.25)	23.0	1807	253 (12.3)
United Kingdom	707 (8.42)	21.9	415	137 (24.8)
Germany	663 (7.89)	12.0	396	104 (20.8)
Canada	609 (7.25)	20.7	341	125 (26.8)
Italy	559 (6.66)	12.5	311	128 (29.2)
China	466 (5.55)	6.7	411	117 (22.2)
Spain	436 (5.19)	11.7	274	55 (16.7)
Australia	429 (5.11)	19.1	228	83 (26.7)
France	355 (4.23)	12.5	206	50 (19.5)
Netherlands	324 (3.86)	19.4	164	57 (25.8)

^a^SCP: single-country publication.

^b^MCP: multiple-country publication.

^c^SCP and MCP were computed by Bibliometrix based on data from the corresponding author’s country only. Hence, their summation did not equal the total number of publications of that country.

**Table 4:** The 10 most productive journals of virtual reality research in medicine (8399 articles).

Journal		Publications, n (%)		Citations per publication (CPP)		2019 Impact Factor		Research area (domain)^a^
Lecture Notes in Computer Science		207 (2.46)		3.2		0.402		Computer science
Cyberpsychology, Behavior, and Social Networking	119 (1.42)		29.3		2.258		Psychology	
Proceedings of SPIE^b^		97 (1.15)		1.6		N/A^c^		Computer science, optics
Surgical Endoscopy and Other Interventional Techniques		96 (1.14)		33.7		3.149		Surgery
Annual Review of Cybertherapy and Telemedicine		92 (1.10)		1.7		N/A		Computer science
PLOS One		81 (0.96)		12.4		2.740		Science and technology
Journal of Neuroengineering and Rehabilitation		73 (0.87)		27.0		3.519		Engineering, neurosciences and neurology, rehabilitation
Studies in Health Technology and Informatics		73 (0.87)		5.8		N/A		Health care sciences and services, medical informatics
Journal of Surgical Education		69 (0.82)		14.7		2.220		Education and educational research, surgery
Frontiers in Psychology		48 (0.57)		7.7		2.067		Psychology

^a^Research area as assigned by Web of Science.

^b^SPIE: Society of Photo-Optical Instrumentation Engineers.

^c^N/A: not available.

When the research area (of journals) was examined, the top 6 areas in descending order of publication count were computer science (CS), engineering (En), neurosciences and neurology (Neuro), surgery (Surg), psychology (Psy), and rehabilitation (Rehab). The CPPs of these 6 groups were significantly (*P*<.001) different from each other. Post-hoc tests revealed that the CPPs of Surg (25.1, SD 40.0), Psy (19.0, SD 35.6), and Neuro (20.3, SD 44.1) were greater than those of En (8.5, SD 26.8) and CS (9.5, SD 76.0); Surg was greater than that of Rehab (12.3, SD 24.5); and Others (15.6, SD 57.5) was greater than those of CS and En. Since some journals can be assigned to multiple research areas, a mutual exclusion procedure was performed in reverse hierarchical order (ie, the Rehab group retained all relevant papers first, then the Psy group retained all relevant papers excluding those coassigned to the Rehab group, then the Surg group retained all relevant papers excluding those coassigned to the Rehab and Psy groups, and so on). Finally, the Others group consisted of papers not assigned to the other 6 groups. In summary, papers dealing with clinical topics of surgery, neurosciences, psychology, and rehabilitation generally had more citations than those dealing with topics of computer science and engineering. This is consistent to a previous study in which neurosciences and surgery were among the top scientific categories with high citations [26].

### Popular Research Themes

**Figure 2:**
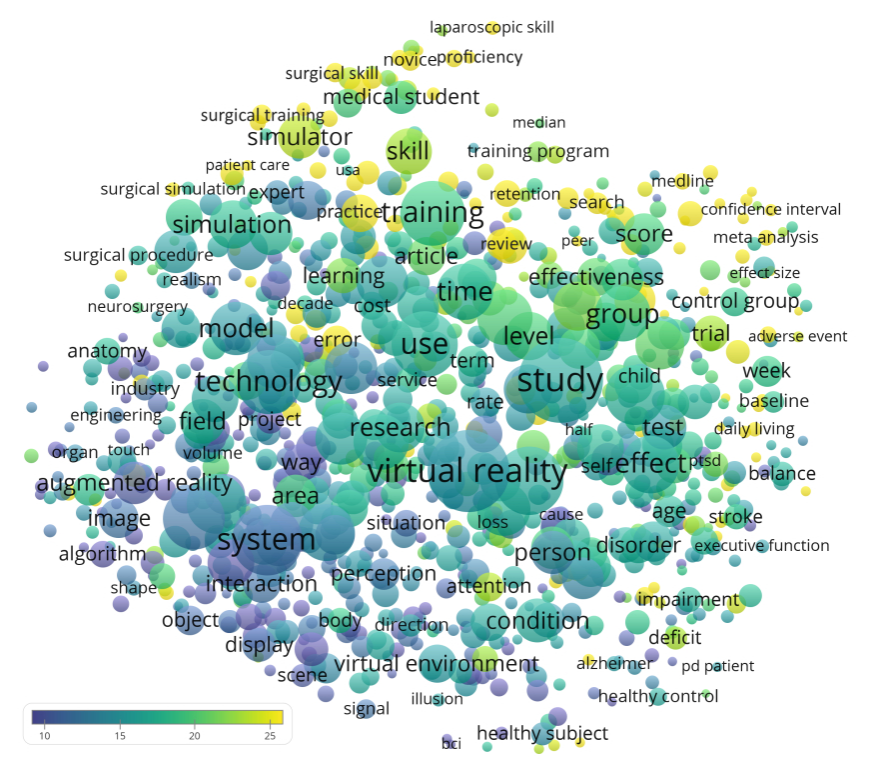
Term map showing phrases from publication titles and abstracts in virtual reality research in medicine. Phrases occurring in at least 0.5% (42/8399) of the publications were included. Multiple appearances in a single publication counted as one. Circle size represents the frequency of occurrence, color represents the citation per publication, and distance between 2 circles represent how 2 phrases co-occurred in the publications.

**Figure 3:**
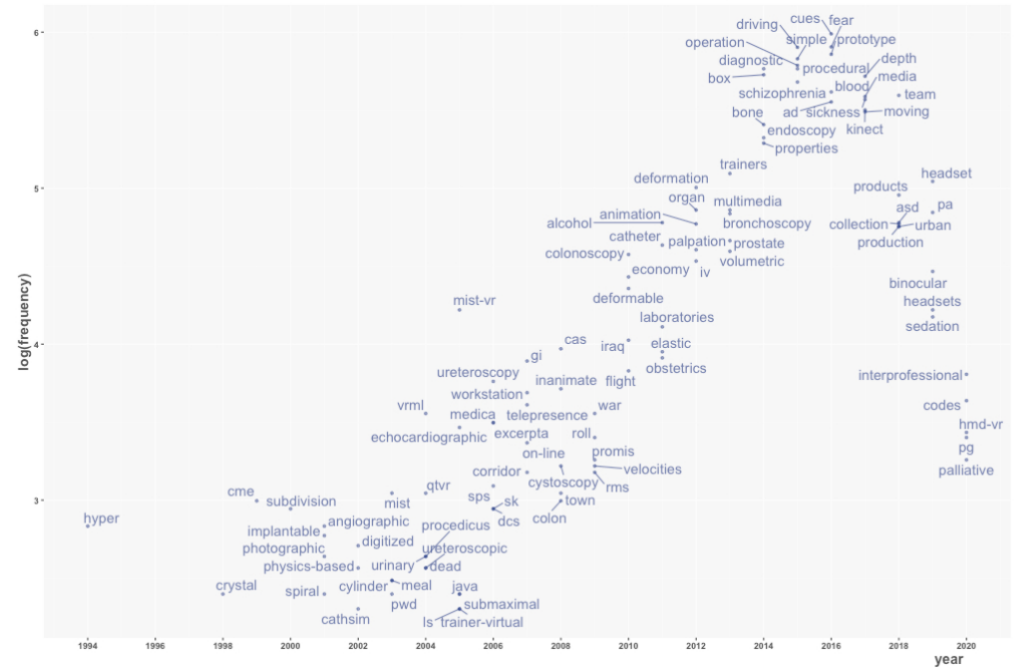
Trends in the abstract words, as determined using the Trend Topics function of Bibliometrix.

VOSviewer was used to generate a term map that tagged the phrases mentioned in the titles and abstracts of the publications with their CPPs (Figure 2). Phrases located in the upper part of the figure generally had higher CPPs. They were mainly related to surgery, such as laparoscopic skill (95/8399, CPP=29.9), surgical skill (190/8399, CPP=35.9), surgical training (195/8399, CPP=29.1), and surgical simulation (103/8399, CPP=26.5). Bibliometrix was used to illustrate the temporal changes in the abstract words (Figure 3). The most common words in the 2000s were related to diagnostic procedures such as angiographic, echocardiographic, ureteroscopy, and colonoscopy. In the 2010s, the most common words were related to brain diseases and mental health disorders, such as schizophrenia, AD (Alzheimer’s disease), and ASD. Table 5 lists the medical conditions mentioned in at least 0.5% (42/8399) of the titles and abstracts of publications of VR research in medicine. This list was enriched with neuropsychological (eg, anxiety, depression), neurophysiological (eg, pain), and neuropathological (eg, Parkinson, Alzheimer) conditions and diseases.

**Table 5:** Medical conditions mentioned in at least 0.5% (42/8399) of the publications of virtual reality research in medicine.

Medical condition	n	Citations per publication (CPP)
Pain	364	18.0
Stroke	319	18.3
Anxiety	316	21.2
Parkinson	258	17.6
Depression	148	18.3
Fear	143	24.7
Cancer	117	13.5
Alzheimer	113	15.1
Dementia	113	14.9
PTSD^a^	94	16.8
Mild cognitive impairment	90	19.7
Cerebral palsy	73	25.8
Traumatic brain injury	67	25.0
Multiple sclerosis	54	13.2
Psychosis	47	20.7
Obesity	46	11.5

^a^PTSD: posttraumatic stress disorder.

**Figure 4:**
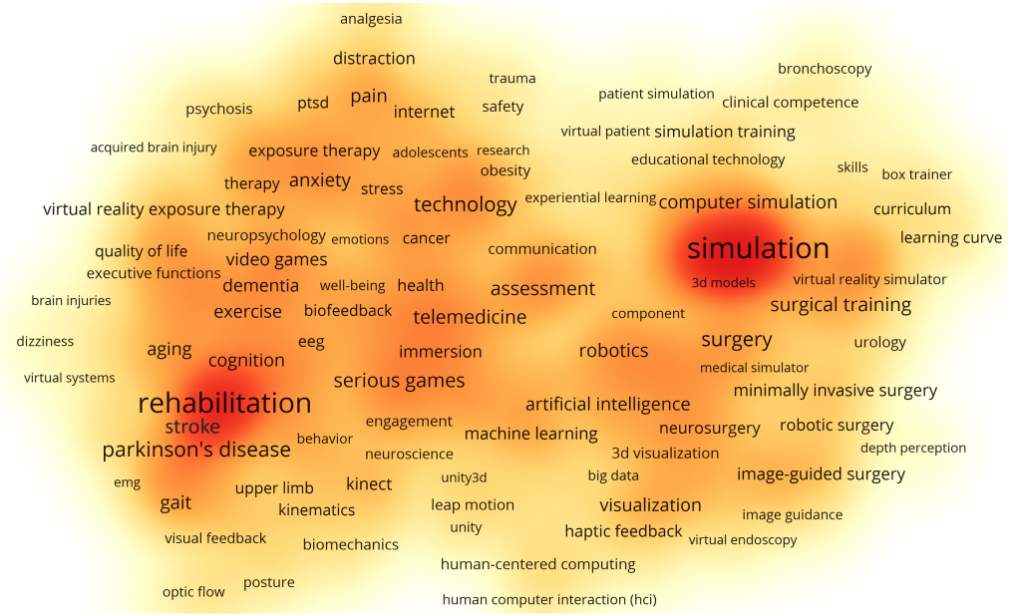
Density map showing author keywords of the publications in virtual reality research in medicine. Keywords occurring in at least 0.1% (9/8399) of the publications were included. The keywords “virtual reality,” “augmented reality,” and “mixed reality” are not shown as they were the major search terms. A keyword or cluster of keywords with higher frequency counts forms a red region, and those with lower frequency counts form a yellow region.

Author keywords also revealed that rehabilitation and simulation were the 2 major foci. As seen in Figure 4, the former was centered by words related to aging and neurodegenerative problems, such as Parkinson’s disease (PD), stroke, dementia, gait, and cognition. The latter was centered by words related to surgery and education, such as surgical training, image-guided surgery, virtual patient, and robotic surgery. There were also other words related to emotion and perception, such as anxiety, stress, and pain, which occupied subclusters with lower frequency densities. The 10 author keywords with the highest frequencies and CPPs are listed in Table 6.

The highest frequency keyword was simulation. Table 7 lists the 10 most cited papers that mentioned “simulation” in their keywords. Besides laparoscopic skills, the use of simulation in general medical education was also covered in terms of implementation issues, availability of products on the market, and recommendations.

**Table 6:** The 10 author keywords with highest frequencies and citations per publication (CPPs; 8399 articles).

Keywords		Publications, n	CPP
**Highest frequency**			
	Simulation	399	19.3
	Rehabilitation	357	13.9
	Education	263	15.9
	Training	259	21.0
	Stroke	211	11.8
	Medical education	209	16.1
	Laparoscopy	145	22.5
	Parkinson’s disease	126	17.1
	Technology	109	9.5
	Surgery	104	18.3
**Highest CPP**			
	Optical flow	9	104.8
	Hemiparesis	9	83.1
	Patient simulation	13	78.0
	Laparoscopic skills	9	74.4
	Research	10	60.4
	Education, medical	9	56.6
	Hemiplegia	10	54.8
	Laparoscopic	9	54.8
	Motor control	21	46.3
	Fear	9	45.2

**Table 7:** The 10 most cited papers with simulation as their keyword.

Authors	Title	Journal	Year	Citations, n
Vassiliou MC, Feldman LS, Andrew CG, Bergman S, Leffondré K, Stanbridge D, Fried GM [27]	A global assessment tool for evaluation of intraoperative laparoscopic skills	American Journal of Surgery	2005	431
Ahlberg G, Enochsson L, Gallagher AG, Hedman L, Hogman C, McClusky III DA, Ramel S, Smith CD, Arvidsson D [28]	Proficiency-based virtual reality training significantly reduces the error rate for residents during their first 10 laparoscopic cholecystectomies	American Journal of Surgery	2007	367
Kneebone R [29]	Simulation in surgical training: educational issues and practical implications	Medical Education	2003	290
Kneebone RL, Scott W, Darzi A, Horrocks M [30]	Simulation and clinical practice: strengthening the relationship.	Medical Education	2004	215
Coles TR, Meglan D, John NW [31]	The role of haptics in medical training simulators: a survey of the state of the art	IEEE Transactions on Haptics	2010	203
Rosen KR [32]	The history of medical simulation	Journal of Critical Care	2008	188
Basdogan C, Ho CH, Srinivasan MA [33]	Virtual environments for medical training: graphical and haptic simulation of laparoscopic common bile duct exploration	IEEE/Asme Transactions on Mechatronics	2001	173
Hassfeld S, Mühling J [34]	Computer assisted oral and maxillofacial surgery–a review and an assessment of technology	International Journal of Oral and Maxillofacial Surgery	2001	164
Vozenilek J, Huff JS, Reznek M, Gordon JA [35]	See one, do one, teach one: advanced technology in medical education	Academic Emergency Medicine	2004	157
Zendejas B, Brydges R, Hamstra SJ, Cook DA [36]	State of the evidence on simulation-based training for laparoscopic surgery: a systematic review	Annals of Surgery	2013	154

The use of AR can be facilitated by many related technologies, for example, wearable tactile sensors [37] and skin-like electronics [38]. In surgery, AR can enhance the surgeon’s vision by offering a virtual transparency of the patient [39]. When learning anatomy, AR can create an illusion that allows the display of internal anatomical structures on the body of the user [40]. These cases illustrate the versatility of AR.

## Discussion

### Principal Findings

This bibliometric analysis of 8399 publications on VR research in medicine revealed that the field began to develop in the 1990s, grew in the 2000s, and has been thriving in the 2010s in terms of both publications and citation counts. Original articles accounted for 63.1% of the literature. The article-to-review ratio was 5.9:1.

The literature had global contributions not only from North America and Europe but also from Asia and Oceania, implying that the application of VR in medicine has attracted worldwide attention. Diagnostic and surgical procedures as well as rehabilitation in neurodegenerative and mental health disorders were major research themes.

The use of VR simulators helped in diagnostic and surgical procedures, such as improving novice hospital residents’ laparoscopic skills in terms of error and procedure time reduction [28]. It was also used to assess the suturing skills of different groups of operators [41]. In surgery, it was suggested that VR and simulation could be of value for 4 aspects, namely training and education, surgical planning, image guidance, and telesurgery [42].

Geriatrics has been exploring the use of VR tasks to assess and train episodic memory in the elderly population, by simulating various environments representative of daily life that cannot be physically replicated in the clinic or rehabilitation centers [43]. The use of VR might also help rehabilitation clinicians conduct telerehabilitation on a remote basis so that the patient (eg, after stroke) carries out exercises at home in a virtual environment and data are then transmitted to the clinician [44]. Moreover, VR could incorporate gamified elements so that the process could be more rewarding (eg, for encouraging patients with PD to do more remotely supervised aerobic exercise) [45]. Nonimmersive VR has also been used to add cognitive challenges and virtual obstacles to treadmill training for older adults, targeting attention, perception, and dual tasking during walking with the aim to reduce fall risk [46]. Since the existing studies were quite diverse, more studies should be conducted to optimize the implementation and evaluate the beneficiary effects in different population groups, so that a recommendation can be made for how to use VR in cognitive rehabilitation [47].

Meanwhile, the use of immersive VR was beneficial in managing a spectrum of emotional problems, such as the fear of heights [48], anxiety disorders [17], depression [49], and ASD [50]. Immersive VR could also reduce pain in patients, such as those with severe burn injuries during wound care [51] and pediatric patients during invasive medical procedures [52,53]. Two functions of VR for this category are distraction [54] and creation of presence [55,56]. Whereas the former distracts the subject from the real-world situation, such as pain-inducing medical procedures, the latter enables the subject to “experience” designed scenarios to facilitate management strategies such as cognitive reappraisal or cognitive behavioral therapy.

To the authors’ knowledge, no previous bibliometric analysis on VR research in the medicine literature overall has been published. A bibliometric analysis of VR research in general (including nonmedical areas) by Cipresso et al [2] similarly revealed the dominance by the United States, United Kingdom, Germany, and China in this research field [2]. Five authors in the top 10 list of this study were also in the top 10 list of Cipresso et al [2], namely Giuseppe Riva, Brenda K. Wiederhold, Albert “Skip” Rizzo, Cristina Botella, and Mariano Alcaniz. This implies that medicine might be among the most important scientific areas for applications of VR in general. The general VR research literature covered broader aspects apart from the research areas reported here and had large shares from mathematical and computational biology, radiology, and social sciences [2]. For VR research in ASD, the United States accounted for 51.2% of all publications, followed by the United Kingdom (16.5%), India, Spain, and China (3%-5% each) [57]. VR research in rehabilitation was similarly led by the United States (29.8%) and the United Kingdom (9.0%), followed by Italy (7.7%), Canada (7.4%), and Germany (5.1%) [58]. Meanwhile, the United Kingdom was most productive in VR research in dementia (43.2%), followed by the United States (39.5%), France (33.5%), Switzerland (28.6%), Germany, Greece, and Italy (23.2% each) [24].

A bibliometric analysis of artificial intelligence research in health and medicine similarly found that the United States was the top contributing country (30.8% versus 29.3% in this VR report) [59]. While China and Italy were the second and third most productive countries in artificial intelligence research in medicine, the United Kingdom did not enter the top 20 list. Here, for VR research in medicine, China and Italy were sixth and fifth on the list, whereas the United Kingdom ranked second. Both fields seemed to have different diseases of interest, but both shared stroke, cancer, AD, and PD [59].

### Limitations

The current work searched one literature database only, implying that some papers could inevitably have been missed. However, as different databases record citation counts differently, it would be impossible to merge data from multiple databases. Moreover, publication and citation counts might not directly reflect the scientific quality of the papers, which might be influenced by various parameters including the sample size, study design, and standard of reporting. These would be best addressed by a systematic review with a focused scope, whereas the current work used bibliometric approach reports on the overall landscape of the literature in the research field.

### Conclusions

Literature in the field of VR research in medicine represented over 8000 publications. The analyzed literature had global contributions with the heaviest contributions from the United States and United Kingdom. More clinically related research areas such as surgery, psychology, neurosciences, and rehabilitation had higher average numbers of citations than computer science and engineering. Diagnostic and surgical procedures and rehabilitation were major research themes. Medical conditions commonly investigated were pain, stroke, anxiety, depression, fear, cancer, and neurodegenerative disorders. The high potential and diversity of applications of VR and AR in medicine are already highly visible, and further improvements in these technologies are expected to both enhance their functionality and make them more accessible to patients, which will finally translate to significant therapeutic or preventive impact.

## Abbreviations

3D: 3-dimensional
AD: Alzheimer’s disease
AR: augmented reality
ASD: autism spectrum disorder
CPP: citation per publication
CS: computer science
En: engineering
Neuro: neurosciences & neurology
PD: Parkinson’s disease
Psy: psychology
Rehab: rehabilitation
Surg: surgery
VR: virtual reality
WoS: Web of Science
